# A content analysis of tobacco and alcohol audio-visual content in a sample of UK reality TV programmes

**DOI:** 10.1093/pubmed/fdz043

**Published:** 2019-06-17

**Authors:** Alexander B Barker, John Britton, Emily Thomson, Abby Hunter, Magdalena Opazo Breton, Rachael L Murray

**Affiliations:** UK Centre for Tobacco and Alcohol Studies, Division of Epidemiology and Public Health, University of Nottingham, Clinical Sciences Building, City Hospital, Nottingham, UK

**Keywords:** alcohol, epidemiology, smoking

## Abstract

**Background:**

Exposure to tobacco and alcohol content in audio-visual media is a risk factor for smoking and alcohol use in young people. We report an analysis of tobacco and alcohol content, and estimates of population exposure to this content, in a sample of reality television programmes broadcast in the UK.

**Methods:**

We used 1-minute interval coding to quantify tobacco and alcohol content in all episodes of five reality TV programmes aired between January and August 2018 (*Celebrity Big Brother*; *Made in Chelsea*; *The Only Way is Essex*; *Geordie Shore* and *Love Island*), and estimated population exposure using viewing data and UK population estimates.

**Results:**

We coded 5219 intervals from 112 episodes. Tobacco content appeared in 110 (2%) intervals in 20 (18%) episodes, and alcohol in 2212 (42%) intervals and in all episodes. The programmes delivered approximately 214 million tobacco gross impressions to the UK population, including 47.37 million to children; and for alcohol, 4.9 billion and 580 million respectively.

**Conclusion:**

Tobacco, and especially alcohol, content is common in reality TV. The popularity of these programmes with young people, and consequent exposure to tobacco and alcohol imagery, represents a potentially major driver of smoking and alcohol consumption.

## Background

In the year to March 2017 in England, smoking and alcohol consumption respectively caused an estimated 484 and 337 thousand hospital admissions,^1,2^ at a cost of £6 billion to the National Health Service and a substantially greater cost to wider society.^3–5^ Since almost all adults who smoke begin smoking during teenage years,^6^ and alcohol consumption in adolescence is associated with a higher risk of consumption in adulthood^7^ it is important to prevent children and adolescents from experimenting with these behaviours. The latest data shows that 38% and 22% of 11–15 year olds in England have had an alcoholic drink and tried smoking respectively.^8^

There is now strong evidence that exposure to advertising or other tobacco or alcohol audio visual content (AVC) in the media, including television programmes, increases tobacco and alcohol use in adolescents.^9–17^ Television programme content is widely seen, since an estimated 28 million British homes have at least one television^18^ and in 2017 the average person watched nearly three and a half hours of television each day.^19^ The Office of Communications (Ofcom) Broadcasting Code^20^ protects under-18s by restricting depictions of tobacco or alcohol use in programmes made for children, and discouraging the glamorization of tobacco or alcohol use in programmes broadcast before the 9 p.m. watershed^21^ or otherwise likely to be widely seen, heard or accessed by children.

However, despite these regulatory controls,^20^ tobacco and alcohol imagery remains prevalent in prime-time television programmes.^22–25^ We have previously demonstrated that reality television shows, although usually broadcast after the 9 p.m. watershed but easily accessible to children through online ‘catch-up’ services, sometimes contain high levels of tobacco and alcohol imagery,^22,26,27^ often more prevalent than programmes broadcast within UK prime-time television viewing times. The amount of content in reality TV programmes is likely to be seen by a large amount of young people, for example, our analysis of the 2017 series of the reality TV programme ‘*Love Island*’ estimated that the series delivered an 559 million gross tobacco impressions to its predominantly young adult audience, including over 47 million impressions to children aged under 16.^26^ Viewing figures for reality TV programmes have increased substantially in recent years,^28^ especially among pre-teen and teenaged viewers^29–33^ and it is possible that this genre of programme is exposing young audiences to tobacco and alcohol content. While, a content analysis of a single show, such as *‘Love Island’* is useful for changing policy,^26,34^ viewers may watch multiple reality TV shows throughout the year and different reality TV shows might appeal to different viewer demographics.^35,36^

This study therefore quantifies tobacco and alcohol AVC in a wider sample of reality TV programmes broadcast on UK television in early 2018, compares these findings with previous analyses of prime-time UK terrestrial television content,^24,25^ and estimates the population exposure that this AVC generates.

## Methods

We defined reality TV programmes as those chronicling people in their daily lives or in fabricated scenarios representing everyday life, and chose five programme series broadcast on a variety of UK television channels aired on UK television between 1 January 2018 and 1 August 2018. Whilst we do not know the target population of viewers for these programmes, the selected programmes are known from previous research or from news reports to be likely to attract younger viewers.^26,27,35,37,38^ Celebrity Big Brother involved celebrities living together isolated from the outside world for a period of time. Made in Chelsea is a structured reality TV programme (parts of the show are scripted or made for television) which follows the lives of affluent young people living in London. The Only Way is Essex is a structured reality TV programme which follows the lives of people living in Essex. Geordie Shore follows housemates’ daily lives as they live together for a number of weeks. Love Island is a dating reality TV programme in which young contestants compete for a £50 000 prize by living in a Spanish villa.

We measured tobacco and alcohol content using one-minute interval coding, a semi-quantitative method used extensively in previous studies,^39–41^ and coding each interval for the presence of alcohol and tobacco content in the following categories, as previously described:^23,26,41,42^

### Actual use

Use of tobacco or alcohol onscreen by any character, such as seeing a person smoke a cigarette or drink from a pint glass of beer.

### Implied use

Any inferred tobacco or alcohol use without any actual use on screen, such as a verbal reference that a person is going to smoke or drink, or a behavioural reference such as removing a cigarette from a packet or holding an alcoholic drink.

### Tobacco paraphernalia/other alcohol reference

The presence onscreen of tobacco or alcohol or related materials, such as a lighter or a beer pump/bottle.

### Brand appearance

The presence of clear and unambiguous tobacco or alcohol branding, such as seeing a brand on a cigarette packet/beer bottle.

Tobacco and alcohol content were recorded as present in the one-minute interval if there was one appearance of any category in that interval. More than one category could be coded in a single interval, for example both alcohol and tobacco use. Multiple instances of the same category in the same interval were recorded as one event, but if the same event overlapped two intervals, this was coded as two separate events. One-third of the recorded footage was coded separately by two authors to ensure accuracy and reliability in the coding method. Coding was completed using Microsoft Excel and, on completion, data were entered into IBM SPSS Statistics 24 for analysis. We compared content with findings of our earlier studies of tobacco and alcohol content in prime-time UK television broadcast in 2015 using chi-square analysis.

We estimated UK audience exposure using viewing data from *Digital.I*^43^ and used UK mid-year population estimates for 2017^44^ combined with numbers of tobacco and alcohol appearances to estimate gross and per capita impressions by age group, using previously reported methods.^26,45,46^ The method involves combining viewership (obtained from viewing figures) with the number of tobacco and alcohol appearances per episode to provide gross impressions, the estimated number of tobacco/alcohol exposures delivered. Dividing gross impressions by population mid-year estimates provided per capita impressions, the estimated number of tobacco/alcohol impressions delivered to each person. Both gross and per capita impressions were computed by age group. Analyses were conducted in IBM SPSS Statistics (V.24) and Microsoft Excel (2013). The confidence level was set to 95%.

## Results

The 112 reality TV episodes included a total of 5219 one-minute coding intervals, ranging in number by programme from 450 (*The Only Way is Essex*) to 2350 (*Love Island)*. For a breakdown of the tobacco/alcohol content seen in each programme, see Supplementary file 1.

### Tobacco

Tobacco content occurred in 110 intervals (2% of all intervals) across 20 episodes (18% of all episodes), most commonly in the category of inferred tobacco use. Almost all tobacco content (98%) occurred in a single reality TV series, *Celebrity Big Brother*, which included cigarette smoking (actual use) in 55% of episodes, inferred tobacco use (typically people holding, but not seen smoking, a cigarette) in 62%, and tobacco paraphernalia (predominantly cigarette packs) in 45% of episodes (Table 1). Tobacco branding was not seen (Table 2).

**Table 1:** Broadcast details about the reality TV programmes included in the content analysis

Programme	Season	Number of episodes	Transmission dates	Age rating^a^	Channel
Celebrity Big Brother	22	29	02/01/2018–02/02/2018	18	5
Made in Chelsea	15	12	12/03/2018–28/05/2018	15	E4
The Only Way is Essex	22	10	25/03/2018–27/05/2018	12	ITV2
Geordie Shore	17	12	15/05/2018–31/07/2018	15	MTV
Love Island	4	49	04/06/2018–30/07/2018	No Age Rating^b^	ITV2

^a^According to the British Board of Film Classification.

^b^Has been previously shown to be popular with young people.^26^

**Table 2:** The number of intervals and episodes containing tobacco and alcohol AVC by reality TV programme

	Celebrity Big Brother		Made in Chelsea		The Only Way is Essex		Geordie Shore		Love Island	
	Number of intervals (percentage of total intervals) (*n* = 1327	Number of episodes (percentage of total episodes) (*n* = 29)	Number of intervals (percentage of total intervals) (*n* = 564)	Number of episodes (percentage of total episodes) (*n* = 12)	Number of intervals (percentage of total intervals) (*n* = 450)	Number of episodes (percentage of total episodes) (*n* = 10)	Number of intervals (percentage of total intervals) (*n* = 528)	Number of episodes (percentage of total episodes) (*n* = 12)	Number of intervals (percentage of total intervals) (*n* = 2350)	Number of episodes (percentage of total episodes) (*n* = 49)
*Tobacco*										
Any tobacco content	110 (8%)	20 (69%)	0 (0%)	0 (0%)	0 (0%)	0 (0%)	2 (<1%)	2 (17%)	0 (0%)	0 (0%)
Actual tobacco use	44 (3%)	16 (55%)	0 (0%)	0 (0%)	0 (0%)	0 (0%)	0 (0%)	0 (0%)	0 (0%)	0 (0%)
Implied tobacco use	69 (5%)	18 (62%)	0 (0%)	0 (0%)	0 (0%)	0 (0%)	0 (0%)	0 (0%)	0 (0%)	0 (0%)
Tobacco paraphernalia	48 (4%)	13 (45%)	0 (0%)	0 (0%)	0 (0%)	0 (0%)	2 (<1%)	2 (17%)	0 (0%)	0 (0%)
Tobacco branding	0 (0%)	0 (0%)	0 (0%)	0 (0%)	0 (0%)	0 (0%)	0 (0%)	0 (0%)	0 (0%)	0 (0%)
*Alcohol*										
Any Alcohol content	365 (28%)	29 (100%)	355 (63%)	12 (100%)	158 (35%)	6 (60%)	300 (57%)	12 (100%)	927 (39%)	49 (100%)
Actual alcohol use	55 (4%)	21 (72%)	283 (50%)	12 (100%)	133 (30%)	6 (60%)	78 (15%)	12 (100%)	293 (12%)	47 (96%)
Implied alcohol use	318 (24%)	29 (100%)	158 (28%)	12 (100%)	61 (14%)	6 (60%)	291 (55%)	12 (100%)	906 (39%)	49 (100%)
Other alcohol reference	166 (13%)	26 (90%)	149 (26%)	12 (100%)	60 (13%)	6 (60%)	136 (26%)	12 (100%)	107 (5%)	25 (51%)
Alcohol branding	1 (<1%)	1 (3%)	7 (1%)	3 (25%)	13 (3%)	5 (50%)	51 (10%)	12 (100%)	1 (<1%)	1 (2%)

### Tobacco impressions

We estimate that the 112 episodes of reality TV delivered 214 million tobacco gross impressions (95% CI 193.74–235.84) to the UK population, including 47.37 million (95% CI 37.63–57.13) to children aged <16. Tobacco impressions per capita were highest (average 5.04 (95%CI 4.63–5.47)) in the 55-64 age group. Children received on average 0.80 (95% CI 0.64–0.97) per capita impressions, and women received on average more per capita impressions than men (4.92 (95% CI 4.51–5.33) and 2.35 (95%CI 2.06–2.63) respectively). For a breakdown of total gross and per capita impressions per episode see Table S2.

### Alcohol

**Fig. 1:**
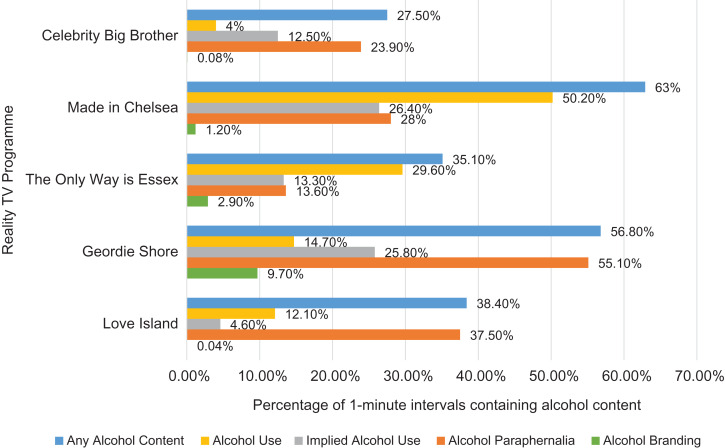
Percentage of one-minute intervals containing alcohol content by programme and coding category.

Alcohol content appeared in all 112 episodes and in 2212 (42%) one-minute intervals, ranging from 28% of intervals in *Celebrity Big Brother* to 63% of those in *Made in Chelsea* (Table 2). The greatest number of intervals including any alcohol content occurred in *Love Island* (Fig. 1).

Actual alcohol use appeared in 925 intervals (18%) across 102 episodes (91%), most commonly involving consumption of wine or champagne (454 intervals, 49% of actual use intervals). Inferred alcohol use was seen in 1767 intervals (34%) across all 112 episodes, most commonly in the form of characters holding alcoholic drinks (1561 intervals, 88%). Alcohol paraphernalia appeared in 653 intervals (13%) across 85 episodes (76%), and typically involved bottles containing alcoholic drinks shown on screen (554 intervals, 85%) (Table 1).

**Fig. 2:**
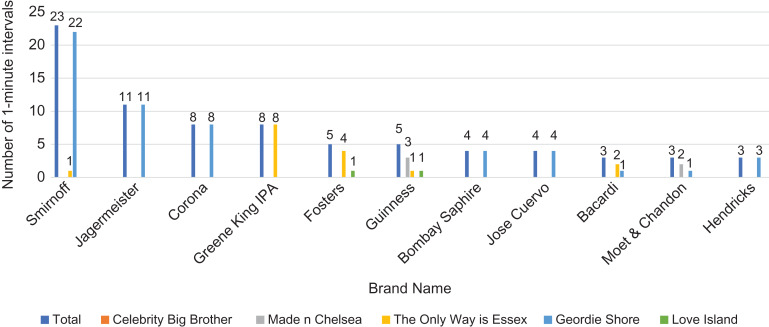
Prevalence of the 10 most commonly observed brands by reality TV programme.

Alcohol branding occurred in 74 intervals (1%) in 23 episodes (21%) across all five programme series, and was most prevalent in *Geordie Shore* (51 intervals, 69% of episodes). Forty different brands were identified, the most common being *Smirnoff* vodka (in 23 intervals), all but one of which occurred in *Geordie Shore.* The second and third most prevalent brands were *Jagermeister* and *Corona*, which occurred exclusively in *Geordie Shore* (Fig. 2).

### Alcohol impressions

We estimate that the 112 episodes of reality TV delivered 4.9 billion alcohol gross impressions (95% CI 4.5–5.3) to the UK population, including 580 million (95% CI 469–690) to children aged <16. Alcohol impressions per capita were highest (average 166.99 (95%CI 157.57–176.41)) in the 16-34 age group. Children received on average 28.19 (95% CI 24.26–158.83) per capita impressions, and women received on average more per capita impressions than men (73.45 (95% CI 66.75–80.15) and (54.35 (95%CI 48.75–59.93) respectively). There were 47 million (95% CI 39.57–283.26) gross impressions of branded alcohol products including 15 million (11.03–19.56) to children. For a breakdown of total gross and per capita impressions per episode see Table S3.

### Comparison with UK terrestrial television content

Comparison with our earlier content analyses of UK prime-time broadcast television^24,25^ demonstrated similar proportions of programmes including any tobacco content, but significantly higher proportions including actual or implied tobacco use, and all categories of alcohol content (Table 3).

**Table 3:** Proportion (%) of broadcasts containing tobacco or alcohol in reality TV and prime-time UK broadcast television

	Reality TV (%)	Prime-time television^23,24^ (%)	*P*-value
Tobacco			
Any tobacco content	18	17	0.76
Actual tobacco use	14	5	<0.01
Implied tobacco use	16	7	<0.01
Tobacco paraphernalia	13	12	0.61
Tobacco branding	0	0.6	0.42
Alcohol			
Any alcohol content	100	54	<0.01
Actual alcohol use	86	11	<0.01
Implied alcohol use	95	38	<0.01
Other alcohol reference	72	40	<0.01
Alcohol branding	20	13	0.04

## Discussion

### Main findings of this study

This study demonstrates that in UK reality TV broadcast in early 2018, tobacco imagery was common in one series (*Celebrity Big Brother*) but occurred rarely or not at all in the other four series analysed. In contrast, alcohol imagery occurred in all 112 episodes we examined. Branding, particularly of three brands (*Smirnoff, Jagermeister* and *Corona*) occurred most commonly in *Geordie Shore.* Comparison with content analysis of prime-time UK television from 2015 demonstrates similar overall levels of tobacco content, but much higher alcohol content. The tobacco and alcohol content in these programmes generates substantial population exposure, of the order of millions of impressions for tobacco, and billions for alcohol. These included millions of tobacco impressions in children aged under 16, and nearly half a billion alcohol impressions.

Our study thus provides evidence that although ostensibly aimed at adult audiences, reality TV programmes in the UK are a significant source of exposure of children to both tobacco and alcohol, but particularly alcohol, imagery. Although our analysis is limited to reality TV programmes produced and broadcast in the UK, the formats of many of these programmes are replicated internationally, making it likely that our findings apply more widely than the UK alone. The five programme series were selected to represent a variety of reality TV programmes, broadcast on a range of free and paid for channels, and all were broadcast after the 9 p.m. watershed.^21^ However these programmes are also likely to appeal to and be watched by children either at the time of broadcast^47^ or at any time of the day on catch up services such as the ITV hub.^48^

Since previous research evaluating the effect of tobacco and alcohol AVC in the media on initiation of tobacco and alcohol use demonstrates an exposure-response relation with exposure to media imagery,^49–51^ our findings indicate that reality TV programmes are a potentially important source of exposure to tobacco and alcohol AVC for children and young adults. We have previously reported high levels of tobacco imagery, including branding, in the 2017 series of ‘*Love Island*’.^26^ That study was initiated in response to newspaper reports of complaints over the level of smoking in the show and demonstrated that whilst the programme makers defended the tobacco content on the grounds of editorial justification the amount of tobacco imagery in the show fell significantly immediately after the public criticism, thus questioning the initial justification. That our present analysis demonstrates that the 2018 series of the show contained no smoking at all, attributed in a newspaper report to an editorial decision,^52^ while achieving greater audience viewing^53^ suggests that the initial justification for smoking content was indeed spurious. Whilst a content analysis of a single show such as *‘Love Island’* is useful for changing policy about tobacco and/or alcohol content shown on TV,^26,34^ viewers may watch multiple reality TV shows throughout the year and different reality TV shows might appeal to different viewer demographics,^35,36^ therefore being exposed to tobacco and alcohol AVC from multiple sources. It is therefore important to keep track of tobacco and alcohol content in programming, and particularly the range of reality TV at aimed at youth audiences^35,36^ so that breaches of policy and/or UK or EU law can be highlighted to regulators.

We have also previously reported high levels of alcohol imagery, and particularly of *Smirnoff* branding, in *Geordie Shore*.^27^ The present study repeats that finding. Whilst a connection has been suggested between MTV and the Smirnoff Brand^27^ it remains unclear whether this branding occurs as a result of any sponsorship or other financial arrangement between the company making *Smirnoff* (Diageo) and the programme makers responsible for *Geordie Shore.* Brands can receive widespread exposure by featuring in television programmes; and whilst the Ofcom Broadcasting Code prevents paid for product placement in the UK,^54^ brands can appear in programmes if they are considered ‘editorially justified’^54^ and were acquired at ‘no significant value’ and no provider has been paid for this (57 p. 52). Alcohol promoters may now therefore be using reality TV programmes to circumvent the Ofcom Broadcasting Code. Love Island formed a financial partnership with Echo Falls wine which resulted in advertising exposure during the advertisement periods of the programme, and the Love Island logo appearing on bottles of Echo Falls. Whilst no Echo Falls branding was apparent in the programme, glasses of wine were observed more regularly than any other type of alcohol in the programme.

Viewing figures for reality TV programmes have increased substantially in recent years,^28^ especially among pre-teen and teenaged viewers.^29–33^ Although section 1.10 of the Ofcom Broadcasting Code states that tobacco and alcohol content ‘must not be condoned, encouraged or glamorized in other programmes likely to be widely seen, heard or accessed by under-eighteens unless there is editorial justification’,^20^ our study suggests that this guidance is not being observed in practice. Further measures may therefore be required to protect under-18s from tobacco and alcohol content in reality TV programmes.

### What is already known on this topic

Initiation of smoking and alcohol use at a young age is a strong risk factor for dependence and continued use in later life. There is strong evidence that exposure to advertising or other tobacco and alcohol imagery in the media increases subsequent use in adolescents. Previous studies have found that alcohol and tobacco content is frequently shown on UK television and that reality TV contains high levels of tobacco and alcohol content (26, 27 2018, Population exposure to smoking and tobacco branding in the UK reality show ‘Love Island’)

### What this study adds

Tobacco and alcohol content shown on TV has an effect on the uptake of smoking and alcohol use in young people. Our analysis shows that reality television programmes are a major source of exposure to young people in the UK and is likely to be a contributor to smoking and alcohol uptake by young people. The Ofcom broadcasting code protects under-18s from tobacco and alcohol content by restricting depictions of tobacco or alcohol use in programmes made for children, and discouraging the glamorization of tobacco or alcohol use in programmes broadcast before the 9 p.m. watershed^21^ or otherwise likely to be widely seen, heard or accessed by children. The current study shows that reality TV programmes, while usually broadcast after the 9 pm watershed, are widely seen and accessed by young people and that this genre of programme is exposing young people to tobacco and alcohol content. Quantifying AVC content can be used to impact policy and change depictions in the media. Tighter scheduling rules from Ofcom, such as restricting the amount of content and branding shown in these programmes, could prevent children and adolescents being exposed to the tobacco and alcohol content found in reality TV programmes.

### Limitations of this study

We coded reality TV programmes which aired during early 2018, this timeframe was chosen to allow the study to be completed in a timely manner, however we acknowledge that reality TV programmes are shown throughout the year. The number of programmes included in the current analysis were chosen for practical reasons, however other reality TV programmes were shown on UK television during this time period. The current study only explored tobacco and alcohol content shown in programmes broadcast on UK television, this did not include content on video-on-demand services. The viewing figures used to estimate exposure to tobacco and alcohol content group viewers into age groups. Whilst we can estimate exposure to this content in under-16’s we do not have information on the number of children at each age who were exposed to this content.

This study has shown that reality TV programmes in the UK are a significant source of exposure of children to both tobacco and alcohol, but particularly alcohol, imagery. Future studies should explore a larger sample of reality TV programmes for a longer period of time and at different points in the year in order to gain a more representative picture of tobacco and alcohol exposure in reality TV programming.

## Supplementary Material

fdz043_Supplementary_File_1_CA_per_programme

fdz043_Reality_TV_supplementary_2

fdz043_Reality_TV_supplementary_file_3

## References

[1] NHS Statistics on Smoking - England 2018, 2018 https://digital.nhs.uk/data-and-information/publications/statistical/statistics-on-smoking/statistics-on-smoking-england-2018/part-1-smoking-related-ill-health-and-mortality.

[2] NHS Statistics on Alcohol - England 2018, 2018 https://digital.nhs.uk/data-and-information/publications/statistical/statistics-on-alcohol/2018/part-1.

[3] Public Health England Alcohol Treatment in England 2013-14. 2014.

[4] Allender S, Balakrishnan R, Scarborough P et al. The burden of smoking- related ill health in the UK. Tob Control 2009;18(4):262. 19509003 10.1136/tc.2008.026294

[5] Callum C, Boyle S, Sandford A Estimating the cost of smoking to the NHS in England and the impact of declining prevalence. HEPL 2011;6(4):489–508. 20735898 10.1017/S1744133110000241

[6] Public Health England Smoking and tobacco: applying all our health 2017 https://www.gov.uk/government/publications/smoking-and-tobacco-applying-all-our-health/smoking-and-tobacco-applying-all-our-health.

[7] Bonomo YA, Bowes G, Coffey C et al. Teenage drinking and the onset of alcohol dependence: a cohort study over 7 years. Addiction 2004;99(1):1520–8. 15585043 10.1111/j.1360-0443.2004.00846.x

[8] HSCIC Smoking, drinking and drug use among young people in England – 2014, 2015.

[9] US Department of Health and National Services Preventing Tobacco Use Among Youth and Young Adults: A Report of the Surgeon General 2012. 22876391

[10] Anderson P, De Brujin A, Angus K et al. Impact of alcohol advertising and media exposure on adolescent alcohol use: a systematic review of longitudinal studies. Alcohol Alcohol 2009;44(3):229–43. 19144976 10.1093/alcalc/agn115

[11] Smith L, Foxcroft DR The effects of alcohol advertising, marketing and portrayal on drinkin behaviour in young people: systematic review of prospective cohort studies. BMC Public Health 2009;9(51):1–11. 19200352 10.1186/1471-2458-9-51 PMC2653035

[12] Hanewinkel R, Sargent JD, Hunt K et al. Portrayal of alcohol consumption in movies and drinking initiation in low-risk adolescents. Pediatrics 2014;133:973–82. 24799536 10.1542/peds.2013-3880 PMC4035596

[13] Chang F, Miao N, Lee C et al. The association of media exposure and media literacy with adolescent alcohol and tobacco use. J Health Psychol 2016;21(4):513–25. 24788103 10.1177/1359105314530451

[14] Leonardi-Bee J, Nderi M, Britton J Smoking in movies and smoking initiation in adolescents: systematic review and meta-analysis. Addiction 2016;111(10):1750–63. 27043456 10.1111/add.13418

[15] U.S. Department of Health and Human Services Preventing Tobacco Use Among Youth and Young Adults: A Report of the Surgeon General. Atlanta, GA: U.S. Department of Health and Human Services, Centers for Disease Control and Prevention, National Center for Chronic Disease Prevention and Health Promotion, Office on Smoking and Health, 2012.

[16] U.S. Department of Health and Human Services The Health Consequences of Smoking: 50 Years of Progress: A Report of the Surgeon General. Atlanta, GA: U.S. Department of Health and Human Services, Centers for Disease Control and Prevention, National Center for Chronic Disease Prevention and Health Promotion, Office on Smoking and Health, 2014.

[17] National Cancer Institute The Role of the Media in Promoting and Reducing Tobacco Use. Tobacco Control Monograph No. 19.: U.S. Department of Health and Human Services, National Institutes of Health; 2008.

[18] Broadcasters’ Audience Research Board Television ownership in private domestic households 1956-2017 2017 http://www.barb.co.uk/resources/tv-ownership/.

[19] Ofcom Scotland leads the UK for time spent watching TV 2018 https://www.ofcom.org.uk/about-ofcom/latest/media/media-releases/2018/scotland-time-watching-tv#1.

[20] Ofcom The Ofcom Broadcasting Code (with the Cross-promotion Code and the On Demand Programme Service Rules) 2017 https://www.ofcom.org.uk/tv-radio-and-on-demand/broadcast-codes/broadcast-code.

[21] Ofcom What is the Watershed? 2013 https://www.ofcom.org.uk/tv-radio-and-on-demand/advice-for-consumers/television/what-is-the-watershed.

[22] Lyons A, McNeill A, Britton J Tobacco Imagery on prime time UK television. Tob Control 2014;23:257–63. 23479113 10.1136/tobaccocontrol-2012-050650 PMC3995275

[23] Lyons A, McNeill A, Britton J Alcohol Imagery on popularly viewed television in the UK. J Public Health 2013;36(3):426–34. 10.1093/pubmed/fdt074 PMC4181421 23929886

[24] Barker AB, Whittamore KH, Britton J et al. Content analysis of tobacco content in UK television. Tob Control 2018 10.1136/tobaccocontrol-2018-054427. PMC6589448 30104409

[25] Barker AB, Whittamore KH, Britton J et al. A content analysis of alcohol content in UK television. J Public Health 2018: fdy142. 10.1093/pubmed/fdy142. PMC6785681 30358860

[26] Barker AB, Opazo Breton M, Cranwell J et al. Population exposure to smoking and tobacco branding in the UK reality show ‘Love Island’. Tob Control 2018;27:709–711. 29437828 10.1136/tobaccocontrol-2017-054125

[27] Lowe E, Britton J, Cranwell J Alcohol content in the ‘hyper-reality’ MTV show ‘Geordie Shore’. Alcohol Alcohol 2018;53(3):337–43. 29365032 10.1093/alcalc/agx116 PMC5913666

[28] TV Licensing Telescope Report. 2011.

[29] Kaiser Family Foundation The Reality of Health: Reality Television and the Public Health. Menlo Park, CA: Henry J. Kaiser Family Foundation, 2006.

[30] Patino A, Kaltcheva VD, Smith MF The appeal of reality television for teen and pre-teen audiences: the power of ‘connectedness’ and psycho-demographics. J Advertising Res 2011;51(1):288–97.

[31] Nielson Nielson 2006 Report on Television. New York: Nielson Media Research, 2006.

[32] Nielson Nielson 2007 Report on Television. New York: Nielson, 2007.

[33] Nielson Nielson 2008 Report on Television. New York: Nielson, 2008.

[34] Daily Mail Love Island bosses ‘ban cigarettes in the villa and garden for 2018 series after a wave of complaints last year’ 2018 http://www.dailymail.co.uk/tvshowbiz/article-5759419/Love-Island-bosses-ban-cigarettes-villa-garden-2018-series.html.

[35] Yougov Social Media & TV audiences 2012 https://yougov.co.uk/news/2012/07/26/social-media-tv-audiences/.

[36] BBC News Big Brother: Who is still watching it? 2017 http://www.bbc.co.uk/news/entertainment-arts-40171096.

[37] ITV Programmes—The Only Way is Essex 2018 https://www.itvmedia.co.uk/programmes/programme-planner/the-only-way-is-essex-rpt.

[38] Huffington Post How Celebrity Big Brother proved that younger viewers aren’t the dimwits they’re perceived to be 2014. https://www.huffingtonpost.co.uk/christian-guiltenane/celebrity-big-brother_b_5826948.html.

[39] Cranwell J, Murray R, Lewis S et al. Adolescents’ exposure to tobacco and alcohol content in YouTube music videos. Addiction 2015;110(4):703–11. 25516167 10.1111/add.12835 PMC4402034

[40] Lyons A Tobacco and alcohol in films and on television/Ailsa Lyons. PhD Thesis. University of Nottingham, 2012.

[41] Lyons A, McNeill A, Britton J Tobacco imagery on prime time UK television. Tob Control 2014;23(3):257–63. 23479113 10.1136/tobaccocontrol-2012-050650 PMC3995275

[42] Barker AB, Britton J, Grant-Braham B et al. Alcohol audio-visual content in formula 1 television broadcasting. BMC Public Health 2018;18:1155. 30285686 10.1186/s12889-018-6068-3 PMC6171320

[43] Digital I Digital.I: a fresh perspective on TV viewing figures, 2018 http://www.digital-i.com.

[44] Office for National Statistics Population estimates for the UK, England and Wales, Scotland and Northern Ireland: Mid 2017 2018 https://www.ons.gov.uk/peoplepopulationandcommunity/populationandmigration/populationestimates/bulletins/annualmidyearpopulationestimates/mid2017.

[45] Cranwell J, Opazo-Breton M, Britton J Adult and adolescent exposure to tobacco and alcohol content in contemporary YouTube music videos in Great Britain: a population estimate. J Epidemiol Community Health 2016;70(5):488–92. 26767404 10.1136/jech-2015-206402 PMC4853525

[46] Sargent JD, Tanski SE, Gibson J Exposure to movie smoking among US adolescents aged 10 to 14 years: a population estimate. Pediatrics 2007;119(5):e1167–76. 17473084 10.1542/peds.2006-2897

[47] TV Licensing Telescope 2014 2014 http://www.tvlicensing.co.uk/ss/Satellite?blobcol=urldata&blobheadername1=content-type&blobheadervalue1=application%2Fpdf&blobkey=id&blobtable=MungoBlobs&blobwhere=1370006402279&ssbinary=true.

[48] ITV ITV Hub 2018 https://www.itv.com/hub/itv.

[49] Morgenstern M, Sargent JD, Engels RCME et al. Smoking in movies and adolescent smoking initiation: longitudinal study in six European countries. Am J Prev Med 2013;44(4):339. 23498098 10.1016/j.amepre.2012.11.037 PMC3616269

[50] Mejia R, Pérez A, Abad‐Vivero EN et al. Exposure to alcohol use in motion pictures and teen drinking in Latin America. Alcohol Clin Exp Res 2016;40(3):631–7. 26857804 10.1111/acer.12986 PMC4775386

[51] Sargent JD, Wills TA, Stoolmiller M et al. Alcohol use in motion pictures and its relation with early-onset teen drinking. J Stud Alcohol 2006;67(1):54–65. 16536129 10.15288/jsa.2006.67.54

[52] Daily Mail Love Island bosses ‘ban cigarettes in the villa and garden for 2018 series after a wave of complaints last year’ 2018 http://www.dailymail.co.uk/tvshowbiz/article-5759419/Love-Island-bosses-ban-cigarettes-villa-garden-2018-series.html.

[53] DiCgital spy Love island 2018 final pulls in biggest ITV2 viewing figures of all time 2018 http://www.digitalspy.com/tv/love-island/news/a862823/love-island-2018-final-ratings-viewing-figures-series-4-itv2/.

[54] Ofcom Broadcasting Code Guidance Notes: Section 9: Commercial References in Television Programming. London: Ofcom, 2011.

