# Corrigendum: A content analysis of tobacco and alcohol audio-visual content in a sample of UK reality TV programmes

**DOI:** 10.1093/pubmed/fdz088

**Published:** 2019-07-23

**Authors:** Alexander B Barker, John Britton, Emily Thomson, Abby Hunter, Magdalena Opazo Breton, Rachael L Murray

**Affiliations:** UK Centre for Tobacco and Alcohol Studies, Division of Epidemiology and Public Health, University of Nottingham, Clinical Sciences Building, City Hospital, Nottingham, UK

In Table 2, some of the entries for alcohol content in *The Only Way is Essex* were incorrect. These have now been corrected.

