# Supplementary material for: A content analysis of tobacco and alcohol audio-visual content in a sample of UK reality TV programmes
Source: J Public Health (Oxf). 2019 Jun 17;42(3):561–9. doi: 10.1093/pubmed/fdz043 (PMC7435217; doi:10.1093/pubmed/fdz043)
Supplement: fdz043_Supplementary_File_1_CA_per_programme [file fdz043_supplementary_file_1_ca_per_programme.docx]

Supplementary File 1 – Content Analysis for each programme

Celebrity Big Brother

The 29 episodes of Celebrity Big brother consisted of 1327 1-minute intervals. Tobacco content was seen in 20 episodes (69% of the total number of episodes), across 110 intervals (8% of the total number of intervals). Tobacco use was seen in 16 episodes (55% of episodes) across 44 intervals (3% of intervals) which all featured cigarette smoking. Tobacco use was often seen in groups around other non-smokers (24 intervals, 55% of tobacco use intervals), by males (40 intervals, 91% of tobacco use intervals). Tobacco paraphernalia was seen in 13 episodes (45% of episodes) across 48 intervals (4% of intervals), and mostly consisted of cigarette packets (46 intervals, 96% of tobacco paraphernalia intervals). Implied tobacco use was seen in 18 episodes (62% of episodes) across 69 intervals (5% of intervals), mostly consisting of non-verbal implied use, such as holding a cigarette (67 intervals, 97% of implied use intervals). Alcohol content was seen in all 29 episodes, across 365 1-minute intervals (28% of all intervals). Alcohol use was seen in 21 episodes (72% of episodes) across 55 intervals (4% of intervals) and mostly consisted of wine/champagne (45 intervals, 82% of alcohol use intervals). Alcohol use was often seen in groups with other non-alcohol users (24 intervals, 44% of alcohol use intervals) by males (30 intervals, 55% of alcohol use intervals). Alcohol paraphernalia was seen in 26 episodes (90% of episodes) in 166 intervals (13% of intervals) and mostly consisted of wine glasses (152 intervals, 92% of alcohol paraphernalia intervals). Implied alcohol use was seen in all 29 episodes across 318 intervals (24% of intervals), with a drink in a person’s hand being the most common occurrence (302 intervals, 95% of implied use intervals). Alcohol branding was seen in a single interval when a character peeled off a plain white ‘Lager’ label to reveal a Becks branded bottle.

Made in Chelsea

The 12 episodes of Made in Chelsea consisted of 564 1-minute intervals. No tobacco content was seen in Made in Chelsea. Alcohol content was seen in all 12 episodes, across 355 1-minute intervals (63% of intervals). Alcohol use was seen in all 12 episodes across 283 intervals (50% of intervals) and mostly consisted of wine/champagne (141 intervals, 50% of alcohol use intervals). Alcohol use was often seen in groups with other alcohol users (274 intervals, 97% of alcohol use intervals) in mixed gender scenes (223 intervals, 79% of alcohol use intervals). Alcohol paraphernalia was seen in all 12 episodes in 149 intervals (26% of intervals) and mostly consisted of beer pumps or glasses (141 intervals, 94% of alcohol paraphernalia intervals). Implied alcohol use was seen in all 12 episodes across 158 intervals (28% of intervals), with a drink in a person’s hand being the most common occurrence (114 intervals, 72% of implied use intervals). Alcohol branding was seen in 3 episodes across 7 intervals, 4 brands were seen with Guinness being the most common (3 intervals).

The Only Way is Essex

The 10 episodes of The Only Way is Essex consisted of 450 1-minute intervals. No tobacco content was seen in The Only Way is Essex. Alcohol content was seen in 6 episodes (60% of episodes), across 158 1-minute intervals (35% of intervals). Alcohol use was seen in 6 episodes (60% of episodes) across 133 intervals (30% of intervals) and mostly consisted of scenes involving multiple types of alcohol being consumed (91 intervals, 68% of alcohol use intervals). Alcohol use was often seen in groups with other alcohol users (128 intervals, 96% of alcohol use intervals) in mixed gender scenes (103 intervals, 77% of alcohol use intervals). Alcohol paraphernalia was seen in 6 episodes (60% of episodes) in 60 intervals (13% of intervals) and mostly consisted of beer pumps or glasses (59 intervals, 98% of paraphernalia intervals). Implied alcohol use was seen in 6 episodes (60% of episodes) across 61 intervals (14% of intervals), with a drink in a person’s hand being the most common occurrence (47 intervals, 77% of implied use intervals). Alcohol branding was seen in 5 episodes across 13 intervals, 19 brands were seen with Fosters being the most common (4 intervals).

Geordie Shore

The 12 episodes of Geordie Shore consisted of 528 1-minute intervals. Tobacco content was seen in two 1-minute intervals, on each occasion this involved a no-smoking sign being shown on screen. Alcohol content was seen in all 12 episodes, across 300 1-minute intervals (57% of intervals). Alcohol use was seen in all 12 episodes across 101 intervals (19% of intervals) and mostly consisted of scenes involving spirits or unknown types of alcohol being consumed (61 intervals, 60% of alcohol use intervals). Alcohol use was often seen in groups with other alcohol users (101 intervals, 100% of alcohol use intervals) by males (48 intervals, 48% of alcohol use intervals). Alcohol paraphernalia was seen in all 12 episodes in 136 intervals (28% of intervals) and mostly consisted of alcohol references such as posters and billboards (81 intervals, 60% of alcohol paraphernalia intervals). Implied alcohol use was seen in all 12 episodes across 291 intervals (55% of intervals), with a drink in a person’s hand being the most common occurrence (201 intervals, 69% of implied use intervals). Alcohol branding was seen in all 12 episodes across 51 intervals (10% of intervals), 48 brands were seen with Smirnoff being the most common (22 intervals).

Love Island

The 49 episodes of Love Island consisted of 2347 1-minute intervals. No tobacco content was seen in Love Island. Alcohol content was seen in 48 episodes (98% of episodes), across 901 1-minute intervals (38% of intervals). Alcohol use was seen in 46 episodes (94% of episodes) across 284 intervals (12% of intervals) and mostly consisted of scenes involving wine/champagne being consumed (214 intervals, 75% of alcohol use scenes). Alcohol use was often seen in groups with other alcohol users (284 intervals, 100% of alcohol use intervals) by females (106 intervals, 37% of alcohol use intervals). Alcohol paraphernalia was seen in 25 episodes (51% of episodes) in 107 intervals (5% of intervals) and mostly consisted of wine glasses (95 intervals, 89% of alcohol paraphernalia intervals). Implied alcohol use was seen in 48 episodes (98% of episodes) across 880 intervals (37% of intervals), with a drink in a person’s hand being the most common occurrence (856 intervals, 97% of implied use intervals)). Alcohol branding was seen in 1 episode across 1 interval, 4 brands were each seen once, Guinness, Fosters, Carlsberg and Aspalls.
