# Supplementary material for: A content analysis of tobacco and alcohol audio-visual content in a sample of UK reality TV programmes
Source: J Public Health (Oxf). 2019 Jun 17;42(3):561–9. doi: 10.1093/pubmed/fdz043 (PMC7435217; doi:10.1093/pubmed/fdz043)
Supplement: fdz043_Reality_TV_supplementary_2 [file fdz043_reality_tv_supplementary_2.docx]

|  | Episode | 1 | 2 | 3 | 4 | 5 | 6 | 7 | 8 | 9 | 10 | 11 | 12 | 13 | 14 | 15 | 16 | 17 | 18 | 19 | 20 | 21 | 22 | 23 | 24 | 25 | 26 | 27 | 28 | 29 | 30 | 31 | 32 | 33 | 34 | 35 | 36 | 37 | 38 | 39 | 40 | 41 | 42 | 43 | 44 | 45 | 46 | 47 | 48 |
| --- | --- | --- | --- | --- | --- | --- | --- | --- | --- | --- | --- | --- | --- | --- | --- | --- | --- | --- | --- | --- | --- | --- | --- | --- | --- | --- | --- | --- | --- | --- | --- | --- | --- | --- | --- | --- | --- | --- | --- | --- | --- | --- | --- | --- | --- | --- | --- | --- | --- |
| Celebrity Big Brother | Proportion viewership (000s) | 0.039 | 0.031 | 0.033 | 0.034 | 0.033 | 0.033 | 0.035 | 0.036 | 0.034 | 0.035 | 0.036 | 0.020 | 0.033 | 0.033 | 0.036 | 0.032 | 0.034 | 0.033 | 0.018 | 0.033 | 0.032 | 0.027 | 0.033 | 0.032 | 0.031 | 0.031 | 0.031 | 0.030 | 0.029 |  |  |  |  |  |  |  |  |  |  |  |  |  |  |  |  |  |  |  |
|  | Gross Impressions (Million) | 0 | 0 | 0 | 0 | 9.75 | 3.98 | 8.31 | 0 | 19.98 | 6.70 | 6.29 | 10.86 | 1.91 | 30.54 | 16.93 | 9.36 | 1.97 | 17.34 | 6.42 | 27.50 | 5.62 | 6.30 | 11.50 | 0 | 0 | 0 | 0 | 0 | 1.69 |  |  |  |  |  |  |  |  |  |  |  |  |  |  |  |  |  |  |  |
|  | Per Capita Impressions | 0 | 0 | 0 | 0 | 0.17 | 0.07 | 0.14 | 0 | 0.34 | 0.11 | 0.11 | 0.19 | 0.03 | 0.52 | 0.28 | 0.16 | 0.03 | 0.30 | 0.11 | 0.47 | 0.10 | 0.11 | 0.20 | 0 | 0 | 0 | 0 | 0 | 0.03 |  |  |  |  |  |  |  |  |  |  |  |  |  |  |  |  |  |  |  |
|  |  |  |  |  |  |  |  |  |  |  |  |  |  |  |  |  |  |  |  |  |  |  |  |  |  |  |  |  |  |  |  |  |  |  |  |  |  |  |  |  |  |  |  |  |  |  |  |  |  |
| Made In Chelsea | Proportion Viewership (000s) | 0.016 | 0.017 | 0.013 | 0.014 | 0.014 | 0.015 | 0.014 | 0.015 | 0.014 | 0.015 | 0.015 | 0.008 |  |  |  |  |  |  |  |  |  |  |  |  |  |  |  |  |  |  |  |  |  |  |  |  |  |  |  |  |  |  |  |  |  |  |  |  |
|  | Gross Impressions (Million) | 0 | 0 | 0 | 0 | 0 | 0 | 0 | 0 | 0 | 0 | 0 | 0 |  |  |  |  |  |  |  |  |  |  |  |  |  |  |  |  |  |  |  |  |  |  |  |  |  |  |  |  |  |  |  |  |  |  |  |  |
|  | Per Capita Impressions | 0 | 0 | 0 | 0 | 0 | 0 | 0 | 0 | 0 | 0 | 0 | 0 |  |  |  |  |  |  |  |  |  |  |  |  |  |  |  |  |  |  |  |  |  |  |  |  |  |  |  |  |  |  |  |  |  |  |  |  |
|  |  |  |  |  |  |  |  |  |  |  |  |  |  |  |  |  |  |  |  |  |  |  |  |  |  |  |  |  |  |  |  |  |  |  |  |  |  |  |  |  |  |  |  |  |  |  |  |  |  |
| The Only Way is Essex | Proportion Viewership (000s) | 0.016 | 0.016 | 0.018 | 0.016 | 0.017 | 0.017 | 0.017 | 0.017 | 0.017 | 0.014 |  |  |  |  |  |  |  |  |  |  |  |  |  |  |  |  |  |  |  |  |  |  |  |  |  |  |  |  |  |  |  |  |  |  |  |  |  |  |
|  | Gross Impressions (Million) | 0 | 0 | 0 | 0 | 0 | 0 | 0 | 0 | 0 | 0 |  |  |  |  |  |  |  |  |  |  |  |  |  |  |  |  |  |  |  |  |  |  |  |  |  |  |  |  |  |  |  |  |  |  |  |  |  |  |
|  | Per Capita Impressions | 0 | 0 | 0 | 0 | 0 | 0 | 0 | 0 | 0 | 0 |  |  |  |  |  |  |  |  |  |  |  |  |  |  |  |  |  |  |  |  |  |  |  |  |  |  |  |  |  |  |  |  |  |  |  |  |  |  |
|  |  |  |  |  |  |  |  |  |  |  |  |  |  |  |  |  |  |  |  |  |  |  |  |  |  |  |  |  |  |  |  |  |  |  |  |  |  |  |  |  |  |  |  |  |  |  |  |  |  |
| Geordie Shore | Proportion Viewership (000s) | 0.007 | 0.008 | 0.008 | 0.009 | 0.008 | 0.007 | 0.008 | 0.009 | 0.007 | 0.007 | 0.007 | 0.007 |  |  |  |  |  |  |  |  |  |  |  |  |  |  |  |  |  |  |  |  |  |  |  |  |  |  |  |  |  |  |  |  |  |  |  |  |
|  | Gross Impressions (Million) | 0 | 0 | 0 | 0 | 0 | 0 | 0 | 0 | 0.42 | 0 | 0.46 | 0 |  |  |  |  |  |  |  |  |  |  |  |  |  |  |  |  |  |  |  |  |  |  |  |  |  |  |  |  |  |  |  |  |  |  |  |  |
|  | Per Capita Impressions | 0 | 0 | 0 | 0 | 0 | 0 | 0 | 0 | 0.007 | 0 | 0.008 | 0 |  |  |  |  |  |  |  |  |  |  |  |  |  |  |  |  |  |  |  |  |  |  |  |  |  |  |  |  |  |  |  |  |  |  |  |  |
|  |  |  |  |  |  |  |  |  |  |  |  |  |  |  |  |  |  |  |  |  |  |  |  |  |  |  |  |  |  |  |  |  |  |  |  |  |  |  |  |  |  |  |  |  |  |  |  |  |  |
| Love Island | Proportion Viewership (000s) | 0.067 | 0.065 | 0.063 | 0.060 | 0.061 | 0.061 | 0.062 | 0.066 | 0.064 | 0.064 | 0.062 | 0.066 | 0.064 | 0.067 | 0.067 | 0.062 | 0.060 | 0.062 | 0.059 | 0.066 | 0.068 | 0.072 | 0.070 | 0.071 | 0.069 | 0.064 | 0.066 | 0.072 | 0.069 | 0.074 | 0.069 | 0.068 | 0.067 | 0.062 | 0.066 | 0.065 | 0.067 | 0.063 | 0.065 | 0.064 | 0.060 | 0.068 | 0.065 | 0.064 | 0.064 | 0.063 | 0.069 | 0.071 |
|  | Gross Impressions (Million) | 0 | 0 | 0 | 0 | 0 | 0 | 0 | 0 | 0 | 0 | 0 | 0 | 0 | 0 | 0 | 0 | 0 | 0 | 0 | 0 | 0 | 0 | 0 | 0 | 0 | 0 | 0 | 0 | 0 | 0 | 0 | 0 | 0 | 0 | 0 | 0 | 0 | 0 | 0 | 0 | 0 | 0 | 0 | 0 | 0 | 0 | 0 | 0 |
|  | Per Capita Impressions | 0 | 0 | 0 | 0 | 0 | 0 | 0 | 0 | 0 | 0 | 0 | 0 | 0 | 0 | 0 | 0 | 0 | 0 | 0 | 0 | 0 | 0 | 0 | 0 | 0 | 0 | 0 | 0 | 0 | 0 | 0 | 0 | 0 | 0 | 0 | 0 | 0 | 0 | 0 | 0 | 0 | 0 | 0 | 0 | 0 | 0 | 0 | 0 |

Table S2: Estimated tobacco audio-visual content exposure per episode
