# Supplementary material for: A content analysis of tobacco and alcohol audio-visual content in a sample of UK reality TV programmes
Source: J Public Health (Oxf). 2019 Jun 17;42(3):561–9. doi: 10.1093/pubmed/fdz043 (PMC7435217; doi:10.1093/pubmed/fdz043)
Supplement: fdz043_Reality_TV_supplementary_file_3 [file fdz043_reality_tv_supplementary_file_3.docx]

|  | Episode | 1 | 2 | 3 | 4 | 5 | 6 | 7 | 8 | 9 | 10 | 11 | 12 | 13 | 14 | 15 | 16 | 17 | 18 | 19 | 20 | 21 | 22 | 23 | 24 | 25 | 26 | 27 | 28 | 29 | 30 | 31 | 32 | 33 | 34 | 35 | 36 | 37 | 38 | 39 | 40 | 41 | 42 | 43 | 44 | 45 | 46 | 47 | 48 |
| --- | --- | --- | --- | --- | --- | --- | --- | --- | --- | --- | --- | --- | --- | --- | --- | --- | --- | --- | --- | --- | --- | --- | --- | --- | --- | --- | --- | --- | --- | --- | --- | --- | --- | --- | --- | --- | --- | --- | --- | --- | --- | --- | --- | --- | --- | --- | --- | --- | --- |
| Celebrity Big Brother | Proportion viewership (000s) | 0.039 | 0.031 | 0.033 | 0.034 | 0.033 | 0.033 | 0.035 | 0.036 | 0.034 | 0.035 | 0.036 | 0.020 | 0.033 | 0.033 | 0.036 | 0.032 | 0.034 | 0.033 | 0.018 | 0.033 | 0.032 | 0.027 | 0.033 | 0.032 | 0.031 | 0.031 | 0.031 | 0.030 | 0.029 |  |  |  |  |  |  |  |  |  |  |  |  |  |  |  |  |  |  |  |
|  | Gross Impressions (Million) | 56.06 | 41.90 | 21.84 | 24.22 | 50.70 | 11.94 | 20.77 | 39.87 | 31.95 | 24.58 | 14.67 | 21.72 | 17.18 | 38.18 | 31.74 | 26.20 | 19.68 | 44.31 | 8.56 | 25.54 | 11.23 | 25.20 | 15.33 | 5.65 | 10.98 | 32.78 | 20.11 | 12.35 | 10.13 |  |  |  |  |  |  |  |  |  |  |  |  |  |  |  |  |  |  |  |
|  | Per Capita Impressions | 0.95 | 0.71 | 0.37 | 0.41 | 0.86 | 0.20 | 0.35 | 0.68 | 0.54 | 0.42 | 0.25 | 0.37 | 0.29 | 0.65 | 0.54 | 0.45 | 0.34 | 0.75 | 0.15 | 0.44 | 0.19 | 0.43 | 0.26 | 0.09 | 0.19 | 0.56 | 0.34 | 0.21 | 0.17 |  |  |  |  |  |  |  |  |  |  |  |  |  |  |  |  |  |  |  |
|  |  |  |  |  |  |  |  |  |  |  |  |  |  |  |  |  |  |  |  |  |  |  |  |  |  |  |  |  |  |  |  |  |  |  |  |  |  |  |  |  |  |  |  |  |  |  |  |  |  |
| Made In Chelsea | Proportion Viewership (000s) | 0.016 | 0.017 | 0.013 | 0.014 | 0.014 | 0.015 | 0.014 | 0.015 | 0.014 | 0.015 | 0.015 | 0.008 |  |  |  |  |  |  |  |  |  |  |  |  |  |  |  |  |  |  |  |  |  |  |  |  |  |  |  |  |  |  |  |  |  |  |  |  |
|  | Gross Impressions (Million) | 28.83 | 22.71 | 22.74 | 28.01 | 20.89 | 25.58 | 27.94 | 23.34 | 19.34 | 22.63 | 22.07 | 24.01 |  |  |  |  |  |  |  |  |  |  |  |  |  |  |  |  |  |  |  |  |  |  |  |  |  |  |  |  |  |  |  |  |  |  |  |  |
|  | Per Capita Impressions | 0.49 | 0.39 | 0.39 | 0.48 | 0.36 | 0.44 | 0.48 | 0.40 | 0.33 | 0.39 | 0.38 | 0.41 |  |  |  |  |  |  |  |  |  |  |  |  |  |  |  |  |  |  |  |  |  |  |  |  |  |  |  |  |  |  |  |  |  |  |  |  |
|  |  |  |  |  |  |  |  |  |  |  |  |  |  |  |  |  |  |  |  |  |  |  |  |  |  |  |  |  |  |  |  |  |  |  |  |  |  |  |  |  |  |  |  |  |  |  |  |  |  |
| The Only Way is Essex | Proportion Viewership (000s) | 0.016 | 0.016 | 0.018 | 0.016 | 0.017 | 0.017 | 0.017 | 0.017 | 0.017 | 0.014 |  |  |  |  |  |  |  |  |  |  |  |  |  |  |  |  |  |  |  |  |  |  |  |  |  |  |  |  |  |  |  |  |  |  |  |  |  |  |
|  | Gross Impressions (Million) | 31.70 | 20.93 | 29.68 | 22.72 | 27.87 | 23.25 | 23.74 | 25.37 | 30.14 | 22.16 |  |  |  |  |  |  |  |  |  |  |  |  |  |  |  |  |  |  |  |  |  |  |  |  |  |  |  |  |  |  |  |  |  |  |  |  |  |  |
|  | Per Capita Impressions | 0.54 | 0.36 | 0.51 | 0.39 | 0.47 | 0.40 | 0.40 | 0.43 | 0.51 | 0.38 |  |  |  |  |  |  |  |  |  |  |  |  |  |  |  |  |  |  |  |  |  |  |  |  |  |  |  |  |  |  |  |  |  |  |  |  |  |  |
|  |  |  |  |  |  |  |  |  |  |  |  |  |  |  |  |  |  |  |  |  |  |  |  |  |  |  |  |  |  |  |  |  |  |  |  |  |  |  |  |  |  |  |  |  |  |  |  |  |  |
| Geordie Shore | Proportion Viewership (000s) | 0.007 | 0.008 | 0.008 | 0.009 | 0.008 | 0.007 | 0.008 | 0.009 | 0.007 | 0.007 | 0.007 | 0.007 |  |  |  |  |  |  |  |  |  |  |  |  |  |  |  |  |  |  |  |  |  |  |  |  |  |  |  |  |  |  |  |  |  |  |  |  |
|  | Gross Impressions (Million) | 13.48 | 13.08 | 10.95 | 12.51 | 11.44 | 11.85 | 11.30 | 8.07 | 11.40 | 9.13 | 10.93 | 12.41 |  |  |  |  |  |  |  |  |  |  |  |  |  |  |  |  |  |  |  |  |  |  |  |  |  |  |  |  |  |  |  |  |  |  |  |  |
|  | Per Capita Impressions | 0.23 | 0.22 | 0.19 | 0.21 | 0.19 | 0.20 | 0.19 | 0.14 | 0.19 | 0.16 | 0.19 | 0.21 |  |  |  |  |  |  |  |  |  |  |  |  |  |  |  |  |  |  |  |  |  |  |  |  |  |  |  |  |  |  |  |  |  |  |  |  |
|  |  |  |  |  |  |  |  |  |  |  |  |  |  |  |  |  |  |  |  |  |  |  |  |  |  |  |  |  |  |  |  |  |  |  |  |  |  |  |  |  |  |  |  |  |  |  |  |  |  |
| Love Island | Proportion Viewership (000s) | 0.067 | 0.065 | 0.063 | 0.060 | 0.061 | 0.061 | 0.062 | 0.066 | 0.064 | 0.064 | 0.062 | 0.066 | 0.064 | 0.067 | 0.067 | 0.062 | 0.060 | 0.062 | 0.059 | 0.066 | 0.068 | 0.072 | 0.070 | 0.071 | 0.069 | 0.064 | 0.066 | 0.072 | 0.069 | 0.074 | 0.069 | 0.068 | 0.067 | 0.062 | 0.066 | 0.065 | 0.067 | 0.063 | 0.065 | 0.064 | 0.060 | 0.068 | 0.065 | 0.064 | 0.064 | 0.063 | 0.069 | 0.071 |
|  | Gross Impressions (Million) | 94.89 | 49.55 | 89.40 | 56.71 | 39.29 | 67.69 | 95.31 | 74.12 | 79.47 | 64.14 | 57.96 | 107.95 | 86.66 | 105.44 | 118.27 | 97.56 | 42.48 | 83.90 | 55.51 | 89.19 | 51.86 | 76.59 | 78.16 | 70.84 | 32.57 | 93.29 | 58.10 | 121.97 | 96.73 | 130.35 | 77.20 | 80.08 | 102.12 | 54.83 | 62.20 | 19.19 | 109.36 | 55.48 | 34.12 | 82.80 | 67.44 | 39.66 | 56.82 | 26.10 | 82.44 | 59.22 | 20.13 | 117.85 |
|  | Per Capita Impressions | 1.62 | 0.84 | 1.52 | 0.97 | 0.67 | 1.07 | 1.62 | 1.26 | 1.35 | 1.09 | 0.99 | 1.84 | 1.48 | 1.80 | 2.01 | 1.66 | 0.72 | 1.43 | 0.95 | 1.52 | 0.88 | 1.30 | 1.33 | 1.21 | 0.55 | 1.59 | 0.99 | 2.08 | 1.65 | 2.22 | 1.32 | 1.36 | 1.74 | 0.93 | 1.06 | 0.33 | 1.86 | 0.95 | 0.58 | 1.41 | 1.07 | 0.68 | 0.97 | 0.44 | 1.40 | 1.01 | 0.34 | 2.01 |

Table S3: Estimated alcohol audio-visual content exposure per episode
